# Impact of source data on the interpretation of contrast-enhanced magnetic resonance angiography of the lower limbs

**DOI:** 10.1186/1756-0500-7-263

**Published:** 2014-04-23

**Authors:** Mark Lewis, Madhavi Venumbaka, Kevin Gill, James Cannon, Allan Clark, Andoni P Toms, Paul N Malcolm

**Affiliations:** 1 Department of Radiology, Norfolk and Norwich University Hospital, Colney Lane, Norwich NR4 7UY, UK; 2 Department of Radiology, Colchester General Hospital, Turner Road, Colchester, Essex CO4 5JL, UK; 3 Department of Radiology, Royal Shrewsbury Hospital, Shrewsbury and Telford Hospital NHS Trust, Mytton Oak Road, Shrewsbury SY3 8XQ, UK; 4 Department of Radiology, Queen Margaret Hospital, Whitefield Road, Dunfermline KY12 OSU, UK; 5 School of Medicine, Health Policy and Practice University of East Anglia, Norwich, UK

**Keywords:** MRA, Venous contamination, Source data

## Abstract

**Background:**

The primary purpose of this study is to examine whether use of source data is effective in increasing the number of arterial segments that can be interpreted from maximum intensity projections of lower limb MR angiograms. Correlation between sites of arterial disease and venous contamination was also measured. Interpretation of source data is performed routinely by radiologists, but the value of this has not been well studied with randomized studies.

**Results:**

The proportion of segments visible above the knee was 87% using maximal intensity projection alone (MIP) and 88% when the MIP was combined with source data. The proportions were 67% for MIP and 72% for MIP plus source data below the knee. There was substantial agreement between presence of arterial disease and venous contamination in the calf and thigh.

**Conclusion:**

The use of source data increases the number of assessable segments, but not individuals, by a statistically significant but small amount (1.2%, p <0.05). This study supports the association between arterial disease and venous contamination.

## Background

Contrast–enhanced magnetic resonance angiography (CE-MRA) of the lower limbs has high sensitivity and specificity and is an alternative to conventional angiography [1,2]. A recent meta-analysis of lower limb MRA indicates a high degree of accuracy in the assessment of stenoses [3]. The prevalence of asymptomatic peripheral arterial disease lies between 3% and 10% in the general population, increasing to 15% to 20% in persons older than 70 years. The incidence of critical limb ischaemia (CLI) is lower. There are approximately between 500 and 1000 new cases of CLI every year in a European or North American population of 1 million [4].

One of the strengths of CE-MRA is that overview maximum intensity projections (MIPs) of the subtracted whole volume source data are automatically created in-line at acquisition time and display the vasculature without overlying structures. The role of source data in interpretation is not well defined. Review of the source data acquired at MRA has been used to improve assessment of the degree of arterial stenoses [5]. The accuracy of vessel diameter assessment from a MIP is dependent on the spatial resolution of the source data [6,7]. Source data can also be important in preventing misinterpretation of artefacts arising from contrast-enhanced MRA of the lower limbs [8].

Another potential use of source data is to assist in the interpretation of arteries obscured by venous contamination on the overview MIPs. Early studies reported venous contamination to be present in 8-20% of studies [1,9,10]. This artefact obscures the arteries on overview MIP images and has limited the acceptability of the technique. Despite improvements in MR scanner performance and MRA techniques during the last decade, venous contamination continues to limit this method. Even with measurement of aorta to lower limb transit times using a timing bolus and the use of sub-systolic thigh compression, venous contamination is not entirely eliminated [11].

The primary purpose of this study was to examine whether use of source data is effective in increasing the number of arterial segments that can be interpreted from MIPs at peripheral MRA.

## Method

This study was performed in compliance with the 2008 Helsinki Declaration [12] with the approval of the Lincolnshire local research ethics committee (protocol 320).

Over a five month period 36 consecutive patients were enrolled into the study. All patients had a history of claudication of less than 100 yards or critical ischaemia (rest pain or tissue loss). The MR exclusion criteria were standard contra-indications to MR imaging. Patients were screened using a questionnaire for pacemaker or defibrillator, heart valves, cerebral aneurysm clips, stents, metallic implants or joints, pain relief patches, electronic devices, metal dentures, shunts, tattoos or permanent eyeliner, hearing aid or cochlear implant, hairpiece, body piercings, operations on head or heart, surgery in the last 3 months, diabetes, renal disease or asthma, bullets or shrapnel, pellets or metallic fragments and metal fragments in the eyes.

Of 36 patients, 4 patients were excluded from MRI scanning at time of consenting. Two had pacemakers, 1 was claustrophobic and 1 patient was judged unfit to give informed consent.

### MR Technique

MRA was performed on a Philips 1.0 T unit (Philips NT, Philips Healthcare, Guildford, UK) with automated stepping table. Patients were positioned on the MR table feet first and the feet supported in a foot rest. A 20G cannula was inserted into the ante-cubital fossa and flushed through 20 cm of connecting tubing with normal saline. After acquisition of planner scans at 3 stations using the in-built body coil, 3D spoiled gradient echo angiography masks were performed. A mask of the lower legs using 2 anterior elements of the phased array surface coil and then masks of the upper legs and finally of the pelvis using the inbuilt body coil were performed. The pelvis was scanned as a breath-hold. The angiographic sequences were repeated during infusion of triple dose (0.3 mmol/kg body weight) of intravenous gadopentetate dimeglumine (Magnevist, Bayer Healthcare Pharmaceuticals, Wayne, NJ). The contrast agent infusion at 1 ml/second was followed by an equal volume of normal saline at 1 ml/second. Imaging was initiated during a bolus tracking MR fluoroscopic sequence when contrast medium was detected at the aortic bifurcation. Breath hold pelvic imaging was followed by upper leg and then calf acquisitions with 2 table movements of 4 seconds each. The total length of the scan including table movement was chosen to enable appropriate spatial resolution but did not exceed 70 seconds. Maximum slice thickness was 2 mm. Acquisition parameters are shown (Table 1).

**Table 1:** Typical acquisition parameters for automatic stepping table peripheral CE-MRA: (Gradients: maximum amplitude 23mT/m, slew rate 17mT/m/ms)

	**Abdomen**	**Thigh**	**Calf**
TR (ms)	7.5	7.5	7.5
TE (ms)	2.3	2.3	2.3
Flip angle	35	35	35
FOV (cm)	430	430	430
Slice thickness (mm)	1.7	1.7	1.7
No. of slices	50	50	50
Frequency encoding	464	464	464
Phase encoding	128	128	128
NEX	1	1	1
Phase FOV	75%	75%	75%
K space ordering	Reverse centric	Low High	Low High
Spatial resolution	0.84×0.84×1.7	0.84×0.84×1.7	0.84×0.84×1.7
Imaging time (sec)	21	21	21

At the time of each study the interval between commencement of intravenous infusion of contrast agent and time of arrival at the aortic bifurcation was recorded.

### Conventional Angiography (CA)

CA was performed on a Philips MD3 unit with 4 F pigtail placed in the distal aorta 2–3 cms above the iliac bifurcation. Pump injection of up to a maximum of 65 mls of intravenous contrast agent (300 mg iodine/ml) was performed at a rate of 8 ml/sec using bolus chase run images. AP views from aortic bifurcation to the foot arch or as far distally as possible. 30 degree oblique views of the pelvis were also performed when indicated.

CA and MRA were performed within 5 days of each other. Patients were invited to participate in the study at time of consultation when conventional angiography was proposed. An information document was provided if patients expressed willingness to participate and formal witnessed consent was obtained prior to MRA study. If radiological intervention was intended, MRA was performed prior to the conventional angiogram.

### Analysis

The conventional angiograms were performed and reported by an interventional radiologist of 6 years’ experience (KG).

Each MRA study was reviewed at a workstation by a radiologist with 4 years’ experience with special interest in performance and interpretation of MRA (PNM).

The lower limb arterial system was divided in to distal aorta, common iliac, external iliac, common femoral, origin of profunda femoris, superficial femoral (upper, middle and lower segments), popliteal artery (above and below knee), common peroneal artery, anterior and posterior tibial and peroneal ateries (proximal and distal segments). Each segment was assessed for both legs.

Visualised segments were scored as ≤50% stenosis or 51-99% stenosis. The arterial segments not visible were described as not seen (NS). For MRA studies a category of ‘not seen because of venous contamination’ (NSVC) was used if non-visualisation was for this reason.

For MRA, the MIP study was reviewed first without reference to source data, but with knowledge of the clinical information.

Following the MIP review, assessment of stenoses and visualization of each segment was repeated using MIP reformats and unsubtracted source data together.

### Statistics

The number of segments visualised by each technique was compared. The number of visualised segments (stenoses of ≤ 50% and 51-99%) both above and below the knee using MIP alone was compared with MIP combined with source data using McNemar’s test for paired proportions. Statistics were run with individual patients assessed as end-points for statistical significance and also as individual arterial segments. Correlation between venous contamination and arterial disease was measured using Cohen’s kappa coefficient of agreement. The mean transit times between patients with venous contamination and patients without venous contamination were documented with descriptive statistics. All analyses were carried out using MedCalc 12.0 (Mariakerke, Belgium).

## Results

Data from 32 patients were obtained. Fourteen patients were male and 18 were female. The mean age was 74 years (range 48 to 89 years). Nine were diabetic.

1184 segments in 32 patients were assessed by CA and MRA. The number of segments above the knee and below the knee joint that were patent (0-50%, 51-99%), NS and NSVC was recorded for both the MIP only and MIP plus source data MRA (Table 2).

**Table 2:** Demonstration of arterial disease by MRA MIP, MRA MIP & base data and CA by segment: kappa coefficient of agreement, using quadratic weighting, between the angiographic and MR measures of stenosis, k = 0.68 (95% confidence intervals: 0.63-0.73)

	**MRA (MIP Only)**				**MRA (MIP& BD)**				**CA**		
	**≤ 50%**	**51-99% Stenosis**	**NS**	**NSVC**	**≤ 50%**	**51-99% Stenosis**	**NS**	**NSVC**	**≤ 50%**	**51-99% Stenosis**	**NS**
	**Stenosis**				**Stenosis**						
Infra-renal aorta	32	0	0	0	32	0	0	0	32	0	0
Common iliac	60	2	2	0	60	1	3	0	62	2	0
Internal iliac-origin	55	4	4	1	55	5	4	0	56	4	4
External iliac	62	1	1	0	63	0	1	0	60	2	2
Common fem	61	1	2	0	61	1	2	0	61	0	3
Profunda fem	56	5	3	0	57	4	3	0	59	4	1
Superficial fem-up1/3	37	8	15	4	42	6	15	1	44	6	14
Superficial fem-mid1/3	35	8	17	4	38	9	15	2	39	7	18
Superficial fem-low1/3	41	11	12	0	41	10	13	0	37	10	17
Popliteal-AK	49	3	12	0	49	3	12	0	43	7	14
Popliteal-BK	55	2	7	0	54	3	7	0	53	2	9
Ant tib-horizontal	44	3	14	3	47	2	12	3	44	6	14
Ant tib-vert-prox 50%	36	3	21	4	38	4	19	3	31	8	25
Ant tib-vert - dist 50%	34	1	24	5	37	1	23	3	30	1	33
Common peroneal	45	5	8	6	45	6	8	5	46	6	12
Post Tib - prox 50%	36	4	21	3	40	6	16	2	34	7	23
Post Tib – dist 50%	27	2	27	8	33	3	22	6	30	1	33
Peroneal - prox 50%	49	3	8	4	50	3	7	4	47	6	11
Peroneal - dist 50%	39	0	16	9	41	0	15	8	49	0	15

NS – Segment not seen. NSVC – Segment not seen because of venous contamination.

CA – Conventional angiography.

The proportion of arterial segments visible is shown (Table 3). The proportion visible above the knee was 87% at MRA (MIP only) and 88% at MRA (MIP & source data). The proportions were 67% for MRA (MIP only) and 72% for MRA (MIP & source data) below the knee. These findings were very similar to the proportions of segments assessable at CA, 88% above and 70% below the knee. The kappa coefficient of agreement, using quadratic weighting, between the angiographic and MR measures of stenosis was k = 0.68 (95% confidence intervals: 0.63-0.73) indicating “substantial” agreement [13].

**Table 3:** Visualisation of arterial segments: Summary for all methods above and below the knee joint

		**≤ 50% Stenosis**	**51-99% Stenosis**	**NS**	**NSVC**	**% of total segments assessable**
**Above knee**	MIP only	488	43	68	9	87%
	MIP/source data	498	39	68	3	88%
	Conv Angio	493	42	73	0	88%
**Below knee**	MIP only	365	23	146	42	67%
	MIP/source data	385	28	129	34	72%
	Conv Angio	364	37	174	0	70%

**Figure 1:**
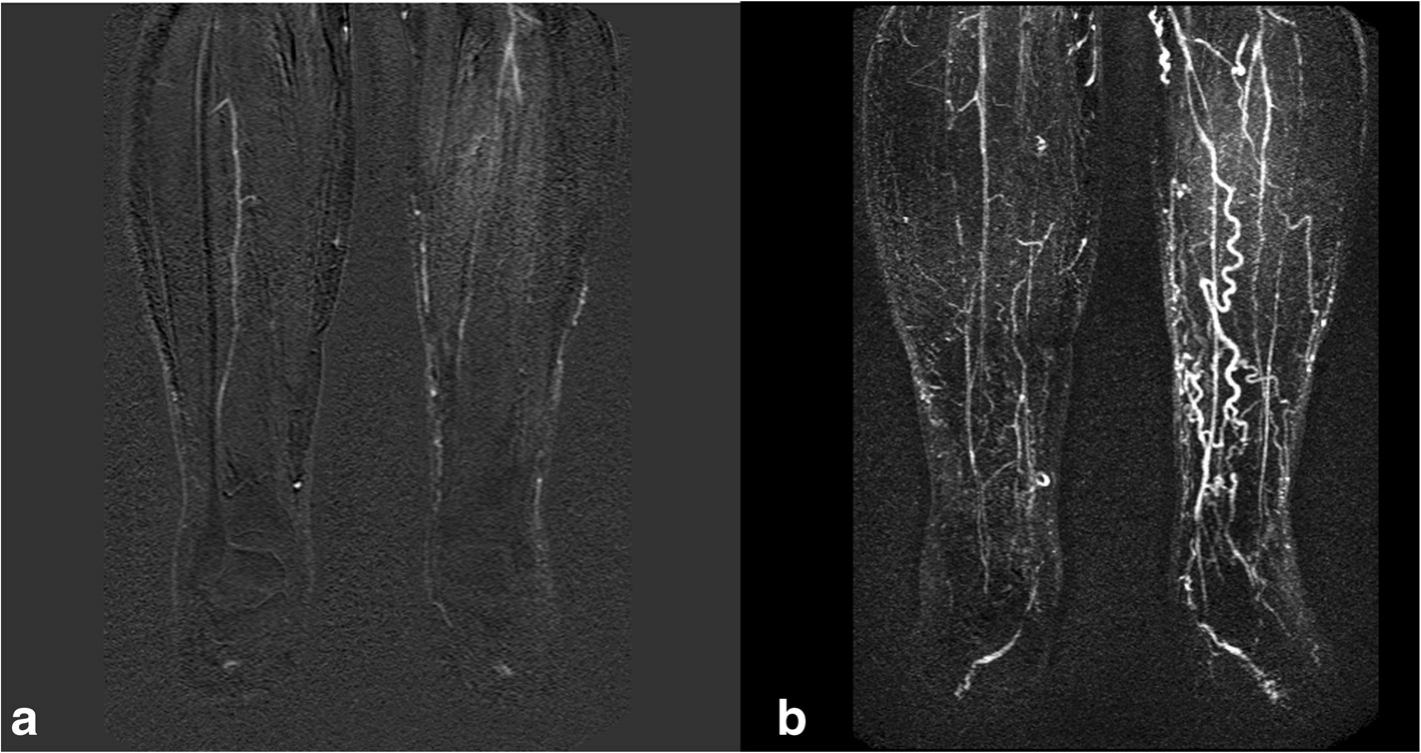
**75 year old male diabetic with dusky feet and ulcer on right foot. (a)** MIP of diseased calf run-off with venous contamination. **(b)** Source data shows the right peroneal artery with greater clarity but the same information was elicited form the MIP.

The proportion of segments not visible because of VC using MIP images alone was 1.5% above the knee and 7.3% below the knee. When both MIP and source data were read, the proportion was 0.5% above the knee and 5.9% below the knee (Figure 1). These differences in proportions interpretable were significant for all segments considered together (P < 0.001) and when below knee segments (P = 0.008) were assessed separately to above the knee (P = 0.03). (Tables 4, 5 and 6). When comparing MIP and MIP/source data readings for an individual patient (n = 32), the differences are not statistically significant (above knee p = 0.06, below knee p = 1.0).

**Table 4:** Contingency table comparing MIP with MIP + source data for all lower limb assessable segments demonstrating a small but significant difference in proportions

**MIP**	**MIP and source data**		**Total**
	**Assessable**	**NSVC**	
Assessable	1133	0	1133
NSVC	14	37	51
Total	1147	37	1184
Difference of proportions (95% confidence intervals)		-0.012 (-0.012 to -0.006)	
Two-tailed p (McNemar)		0.0001	

**Table 5:** Contingency table comparing MIP with MIP + source data for assessable segments above the knee demonstrating no significant difference in proportions

	**MIP and source data**		**Total**
**MIP**	**Assessable**	**NSVC**	
Assessable	599	0	599
NSVC	6	3	9
Total	605	3	608
Difference in proportions (95% confidence intervals)		-0.010 (-0.010 to -0.001)	
Two-tailed p (McNemar)		0.031	

**Table 6:** Contingency table comparing MIP with MIP + source data for assessable segments below the knee demonstrating a small but significant difference in proportions

	**MIP and source data**		**Total**
MIP	Assessable	NSVC	
Assessable	534	0	534
NSVC	8	34	42
Total	542	34	576
Difference of proportions (95% confidence intervals)		-0.014 (-0.014 to -0.004)	
Two-tailed p (McNemar)		0.008	

Sites of venous contamination were compared with sites of arterial disease and the transit time from start of injection to the iliac arteries. Venous contamination and arterial disease were most prevalent in the calf segments, where they affected 63% and 75% of segments respectively. The pelvic segments were least affected, with VC only visible in one segment (3.1%) and arterial disease present in three segments (9.4%) (Table 7). Cohen’s kappa coefficient of agreement between the presence of arterial disease and VC was κ = 0.69 (95% CI: 0.55-0.83) which corresponds to “substantial agreement” [13].

**Table 7:** Contingency tables describing the relationship between venous contamination and arterial disease in the pelvis, thigh and calf

**Region**	**Diseased**	**VC**	**No VC**	κ*	**95% confidence intervals**
Calf	Yes	19	5	0.57	(0.28-0.87)
	No	1	7		
Thigh	Yes	20	5	0.446	(0.10-0.79)
	No	2	5		
Pelvis	Yes	1	2	0.48	(-0.12-1.0)
	No	0	29		
All	Yes	40	12	0.69	(0.55-0.83)
	No	3	41		

*Cohen’s Kappa Agreement Co-efficient.

**Figure 2:**
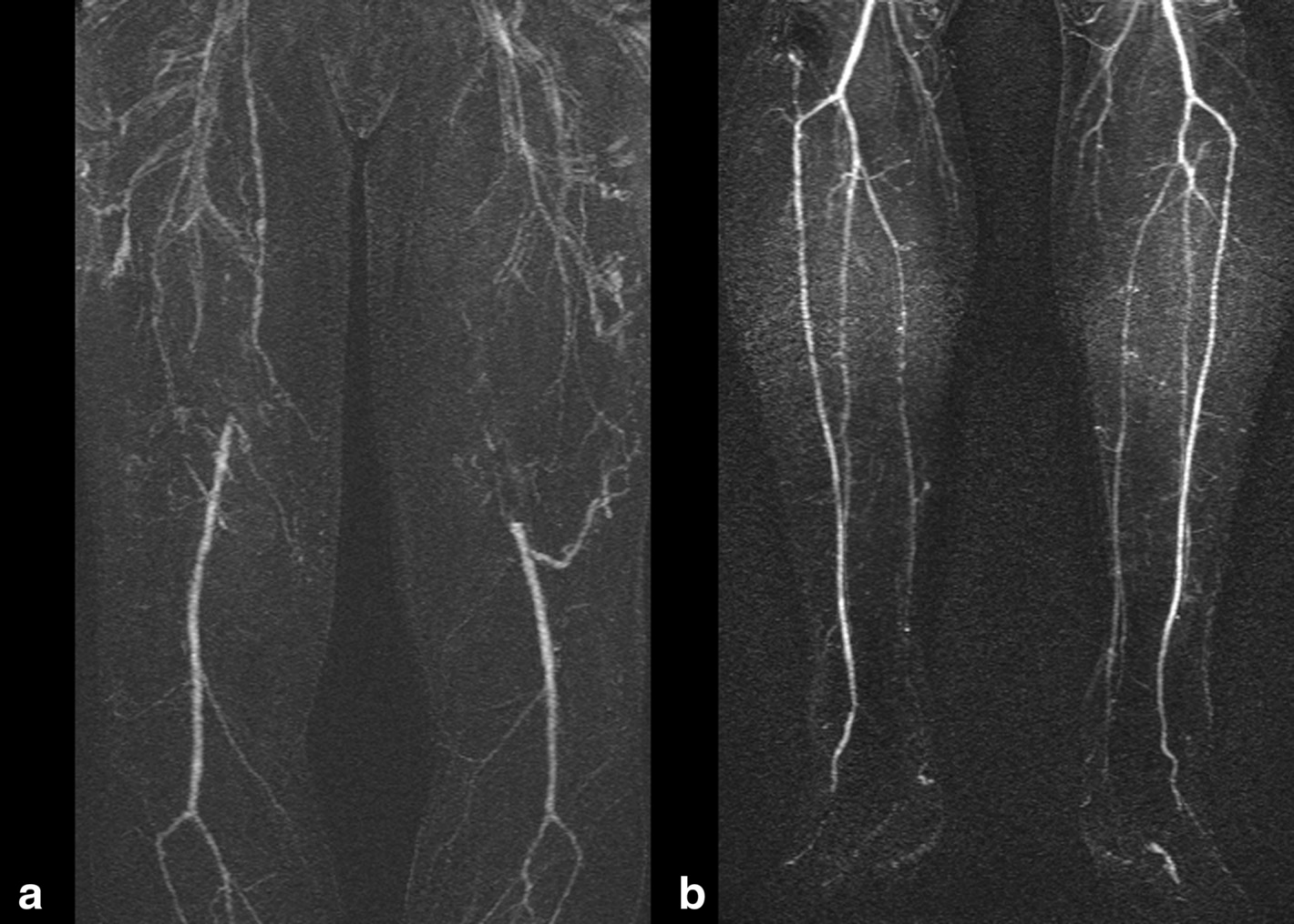
**75 year old male diabetic with bilateral calf claudication. (a)** Superficial femoral disease with venous contamination **(b)** Three vessel run off and absence of venous contamination in the calf.

The transit time for patients with VC (n = 28) was 22.1 s (SD 4.1) and the mean transit time for patient with no VC (n = 4) was 29.25 s (SD 6.5). The small numbers of patients with no VC meant that no meaningful statistical comparison was possible (Figure 2).

## Discussion

Since the introduction of contrast-enhanced MRA of the lower limbs, there have been several advances which have reduced the severity of venous contamination. Increases in gradient strength and multi-element array coils have enabled more rapid image acquisition, with greater signal and spatial resolution. Parallel imaging has also reduced acquisition times reducing venous contamination [14].

Successful alternative strategies to optimize the time of scanning of the calf and so reduce venous contamination in the calf have been used. These include bolus timing injection to predict the interval between starting infusion and arrival time at the calf [15], or the use of dual injection techniques with scanning of the calf before the upper stations [16]. Time resolved imaging of the calf [17] or the use of subsystolic thigh compression which increases the arterio-venous transit time [18,19] have also been useful. Advancing technologies such as high field strength and multi-element receiver coils substantially reduce the drawbacks of MRA and have generated a resurgence of interest in non-contrast MRA [20,21]. However even the use of a timing bolus, thigh compression and optimization of scan parameters on state of the art hardware do not entirely eliminate venous contamination during 3 station bolus chase acquisition [11]. The use of blood pool contrast agents is currently limited by cost and availability but these agents prolong the intravascular residence time. This enables higher resolution scanning which reduces partial volume effects and improves separation of arterial and venous structures on base data [22].

This study has focused on the value of reviewing the source data but additionally demonstrated that CE-MRA at 1.0 T was able to assess an equivalent number of arterial segments to CA, both with and without the use of source data. There was also substantial agreement for the assessment of stenoses for the two techniques.

It is evident that venous contamination will obscure arterial signal on MIP images because these structures are adjacent and may be superimposed. However venous contamination can make visualization of the arteries difficult even on base data because the strongest predictor of venous contamination is arterial disease [14] and so venous contamination is likely to occur when the arteries are most difficult to see, because of local atheromatous disease or poor flow because of more proximal disease. Diabetes mellitus, cellulitis and osteomyelitis result in faster flow to the calf [15], and it is proposed that inflammatory processes increase the speed of arterio-venous transit by reducing arteriolar resistance [14]. This effect is magnified in this series because of the relative severity of disease in this group. All patients had a history of claudication of less than 100 yards or critical ischaemia (rest pain or tissue loss). Poor run-off resulted in segments that could not be assessed at either CA or MRA.

In this study there is substantial agreement between the angiographic, MIP only and MIP/BD techniques for assessment of stenosis/occlusion. More arterial segments are seen when viewing MIP/source data compared with MIP only. Statistical significance (p <0.05) was found both above and below the knee, although the proportion of segments overall affected was small.

While the difference in proportions for all segments is statistically significant we do not have good evidence that routine use of base data is required for increasing the number of visualized segments or to improve stenosis assessment. MR technology also continues to evolve rapidly and any advantage to use of source data in this context is likely to fall with increasing spatial resolution and improving techniques for reduction of venous contamination. Novel MRA techniques, such as electrocardiographically-triggered non-contrast-enhanced magnetic resonance angiography (balanced 3D steady state free precession imaging) may yet finally overcome the effect of venous contamination [23]. Alternatively use of a phase-contrast sequence to determine the flow velocities prior to the acquisition of systolic and diastolic spin echo images can provide subtraction arterial images.

There is data to indicate that the likelihood of venous contamination is correlated with some characteristics of the study population which determine the arterio-venous window. This is the time between first visualisation of arterial contrast and the first visualization of venous return in the lower limb [24]. Venous contamination appears to be a consequence of arterial disease resulting in abnormal arterio-venous transit. This is most commonly seen on more delayed scans of the calf in moving table studies. It is due primarily to shortened arterio-venous transit time in the presence of arterial disease [24] and is compounded by the rapid transit time between common femoral artery and ankle because timing of initiation of infusion, rate of the contrast agent infusion and scan timing at each station using the stepping table technique are not always optimal [25].

This study supports the relationship between arterial disease and venous contamination in the calf and thigh. The lack of significant relationship in the pelvis is likely to reflect the small number of diseased segments (4/1184).

A limitation of this study is the review of CA and MRA data by two single readers. However evidence from recent meta-analysis of peripheral MRA studies shows strong correlation between readers [3] suggesting that this limitation is not likely to affect the findings.

## Conclusions

In conclusion, venous contamination affects a proportion of arterial segments at CE-MRA. The number of segments that can be assessed by review of the source data is greater and this is statistically significant when considering individual arterial segments, but not when comparing individuals. Though use of base data may be important in interpreting artefacts, there is no evidence from this study to suggest that routine review of base data is required for patency or degrees of stenosis when interpretating contrast enhanced MRA of the peripheral arteries. There is a significant relationship between the anatomical level of arterial disease and venous contamination, strengthening the suggestion of a causal relationship as previous studies have suggested.

## Abbreviations

MRI: Magnetic resonance imaging; VC: Venous contamination; MIP: Maximum intensity projection; CA: Conventional angiography; SFA: Superficial femoral artery; AK: Above knee; BK: Below knee.

## Competing interests

The authors declare that they have no competing interests.

## Author’s contributions

ML and MV collated the data, performed the relevant literature search and prepared the manuscript. KG and performed and reported the conventional angiography. JC performed the MR studies and recorded the data. AC and APT provided statistical evaluation. PNM read the MRA and was chief investigator. All authors read and approved the final manuscript.
